# Graph Search-Based Exploration Method Using a Frontier-Graph Structure for Mobile Robots

**DOI:** 10.3390/s20216270

**Published:** 2020-11-03

**Authors:** Hyejeong Ryu

**Affiliations:** Department of Mechatronics Engineering, Kangwon National University, Chuncheon KR24341, Korea; hjryu@kangwon.ac.kr; Tel.: +82-33-250-6376

**Keywords:** mobile robots, exploration, frontier detection, breadth-first search, depth-first search, loop-closing

## Abstract

This paper describes a graph search-based exploration method. Segmented frontier nodes and their relative transformations constitute a frontier-graph structure. Frontier detection and segmentation are performed using local grid maps of adjacent nodes. The proposed frontier-graph structure can systematically manage local information according to the exploration state and overcome the problem caused by updating a single global grid map. The robot selects the next target using breadth-first search (BFS) exploration of the frontier-graph. The BFS exploration is improved to generate an efficient loop-closing sequence between adjacent nodes. We verify that our BFS-based exploration method can gradually extend the frontier-graph structure and efficiently map the entire environment, regardless of the starting position.

## 1. Introduction

Mapping is one of the most critical prerequisites for mobile robots performing various high-level tasks. Simultaneous localization and mapping (SLAM) algorithms construct maps of the environment around a robot while localizing the robot at the same time using continuously received sensor data [1,2,3]. For two decades, many researchers have developed various SLAM algorithms to improve the accuracy of SLAM states in terms of global consistency under the accumulated sensor error caused by large motion sequences. However, SLAM does not select the next target destination for efficient mapping, and only focuses on estimating the state of the robot.

Classic exploration methods find a path that increases information about the environment [4,5,6]. They rely on detecting frontier areas, edges between empty and unknown/unexplored areas. Because these are usually at the limit of the sensors’ range, the robot has a higher probability of obtaining new information about unexplored areas at frontiers than at non-frontier positions. The purpose of frontier-based exploration methods is full coverage of the environment, rather than accuracy of the mapping and localization states.

Instead of considering the mapping coverage, active localization algorithms increase localization accuracy by only calculating a target position within the previously constructed map [7,8]. Active localization algorithms can be combined with path planning algorithms, and are suitable for maintaining the uncertainty of the localization state below a certain level while the robot heads towards its destination.

Integrated exploration, also known as active SLAM, plans a path to improve localization accuracy and increase mapping coverage in an unknown environment [9]. Most integrated exploration algorithms employ the concept of information gain to select the next target candidates for localization and mapping [10,11] and define a cost function based on the information gain to choose one target position. The final result of the integrated exploration algorithm should be an accurate map of the entire environment. Many integrated exploration algorithms detect frontiers using a grid map to guarantee the mapping coverage. The frontier areas can also be detected on dense point cloud maps or continuous representations for environments. Detecting frontier areas and inspecting the mapping coverage can be easy from a grid map because mapped empty areas and unmapped areas are clearly distinguished on grid maps. Although the basic SLAM framework of integrated exploration constructs a feature map or a pose graph for state estimation, a grid map is also produced whenever the robot needs to detect frontiers [12,13]. At this time, the estimated trajectory and corresponding measurements at each pose are used to construct one global grid map.

This paper presents an integrated exploration method that constructs a frontier-graph structure using a local grid map and selects the next exploration target based on this frontier-graph. We address the exploration problems in two-dimensional (2D) environments. The proposed frontier-graph is based on detecting frontier information on grid maps. If we define a frontier area in three-dimensional (3D) representations, the proposed approach can be extended to 3D exploration problems. But, in this paper, we think over 2D exploration problems. The previous integrated exploration approaches that maintain one global grid map can suffer from decreasing efficiency due to the growing size of the map as exploration proceeds. To overcome this, we propose a method that uses several local maps assigned to frontier positions. Unlike most previous approaches that use extracted frontier information to decide just the next target, and do not manage them systematically for further exploration steps, we construct a frontier-graph structure from the frontier nodes on each local map. The frontier-graph structure shows the exploration priority of the adjacent frontier nodes, and we can obtain a loop-closing path, as necessary for the global consistency of SLAM. Therefore, the loop-closure of the SLAM algorithms can be induced by the proposed exploration approach.

The remainder of this paper is organized as follows. Section 2 summarizes related work. In Section 3, we present the frontier-graph structure constructed by the frontier cell segmentation using a local grid map. In Section 4, we present an improved graph-search-based exploration method, breadth-first search exploration, to select the next target node. Section 5 shows simulation results in a well-known benchmark environment to compare the efficiency of two graph-search-based exploration methods. In Section 6, we present our conclusions.

## 2. Related Work

Many integrated exploration algorithms detect frontier areas on grid maps to complete mapping for the whole environment. Several works maintain only a feature map and do not depend on the grid map [14,15,16]. In these cases, their target environments are usually simple, and the purpose is to achieve accurate state estimation rather than full coverage.

Frontier-based integrated exploration algorithms repeat the following three steps until no frontier cells are detected on a global grid map: identifying frontier cells, evaluating the frontier cells using a defined utility function, and selecting the next target. Researchers have mainly studied how to define an appropriate utility or cost function to evaluate the localization and mapping uncertainty reduction at each frontier area. A balanced utility function, which independently calculates the localization uncertainty based on a feature-based map and expected information gain on a grid map, was proposed in [17]. In this approach, an Extended Kalman Filter (EKF) is used for the SLAM estimation problem.

An entropy-based utility function that calculates the joint map and path entropy decrease has also been proposed [10,18]. One study [10] used a Rao-Blackwellized particle filter (RBPF) to estimate and represent the posterior of the map and the robot’s trajectory. Unlike the EKF, the RBPF can be performed in not only feature-based maps, also grid maps. Only one type of map representation, the grid map, was used for both SLAM estimation and frontier detection. The expected information gain along each action sequence for candidate frontier positions was computed by generating the expected measurement on the grid map. Another study [18] employed a pose graph-SLAM as the estimation framework. The pose graph-SLAM maintains the robot’s pose trajectory in the form of a sparse graph [19,20]. It optimizes the graph using relative motion constraints that connect the pose nodes via sensory data. Because the graph-SLAM algorithm does not use a grid representation for state estimation, two studies [11,18] constructed a global grid map using the mean pose of the node in the graph, then used the corresponding raw sensor data or log-odds of the grid map to extract the frontier information.

In the above grid map-based exploration methods, each grid cell shows the binary occupancy information of the corresponding position in the environment. In two studies [21,22], a Gaussian processes (GPs) occupancy map was constructed, with each cell of the GP map containing a continuous occupancy probability. Using GPs’ high-dimensional map inference and a mutual information-based greedy exploration strategy, one of these studies [21] completed mapping for the entire environment with fewer observations than standard binary grid mapping. To learn GP parameters, a study [22] applied a Rapidly Exploring Random Tree (RRT) search guiding a robot towards unvisited informative locations.

Recently, deep learning approaches have been applied to the selection of the next-best-view position on the grid map during integrated exploration. One study [23] proposed an exploration architecture that uses a deep reinforcement learning model to integrate common knowledge in office floor plans. Another study [24] used a deep neural network to predict a robot’s most informative exploratory action.

Most of the methods mentioned above focused on two actions [25]. The first is exploration to select the most informative frontier position on a global grid map constructed through accumulated sensor data until the time of the decision. The aim of this action is to increase the robot’s knowledge of the environment. The second action is exploration that leads the robot to the already mapped areas to reduce the SLAM state uncertainty through active loop-closing. These actions are evaluated on the current global grid map. As the exploration proceeds, the size of the global grid map increases. The growing size of the global map means that the number of candidate positions or actions to be evaluated becomes large. This affects the overall exploration efficiency for achieving full coverage, because frontier-based exploration continues until there is no frontier on the global grid map. The Wavefront Frontier Detector (WFD) and Fast Frontier Detector (FFD) were proposed to avoid processing all the data characterizing the growing grid map [26]. WFD is a graph-search-based algorithm that evaluates only known/observed areas. FFD examines only newly obtained laser measurements to detect frontier cells.

The conventional integrated exploration approaches select the next exploration target from the newly detected frontier regions and previously visited regions on the robot’s trajectory. These next target candidates are re-calculated for each decision, but they are not systematically managed for further decisions. Moreover, the efficiency of frontier-based exploration algorithms depends on the frontier information on the grid map. The global grid map used for detecting frontier regions is continuously merged with the current sensor data based on the current robot position obtained from the SLAM algorithm. The resulting global grid map and its frontier information are affected by the SLAM state’s accuracy. Prior to loop-closing, the uncertainty of the SLAM estimation becomes larger as the travel distance increases [3,27]. In general, it is difficult to correct the occupancy information using a single global grid map unless the robot accesses all the past trajectories and corresponding sensor measurements. In terms of efficient exploration, we need a systematic map updating strategy when the robot’s pose accuracy has been recovered by loop-closing.

Local map-based exploration methods have previously been proposed [28,29] to overcome the inefficiencies that arise due to dependence on one global grid map for detecting frontier cells and to systematically organize the target candidates. In local map-based exploration, the frontier cells are detected from a local map constructed based on current sensor measurements, not from the global grid map. A representative cell is selected among the local frontier cells, and the representative cell and local map are registered to the frontier-tree structure as a node. The relative transformation between the frontier nodes becomes the edge of the frontier-tree. We can efficiently extract real frontier cells, which are frontiers in both the current local map and the local maps of the adjacent nodes, using the frontier-tree structure and map merging process. Moreover, we can select the next target node of the frontier-tree structure according to the exploration priority using two graph search-based exploration methods, depth-first search (DFS) exploration or breadth-first search (BFS) exploration, i.e., a loop-closing event can be induced efficiently using frontier-tree structure. If global consistency of the edge between the frontier nodes is achieved by loop-closing, the local maps of the corresponding loop nodes are merged using the corrected edge information. The local maps of the loop nodes are updated to the merged map.

In this paper, we extend these works in three respects. First, we refine the database of the frontier-graph structure by focusing on the integrated exploration itself, and improve a method for segmenting frontier cells. Segmenting the frontier cells and calculating the frontier node of each segment is crucial to the exploration efficiency. Second, we present a method to register frontier nodes that makes an efficient loop-closing sequence. Furthermore, we compare BFS exploration and DFS exploration using a publicly available environment. This paper assumes that a robot has a 360${}^{\circ }$ scanning range sensor to obtain range measurements for all directions. It means that we do not consider the issues about sensing coverage according to the robot’s orientation. The proposed exploration approach decides the next target position but not the robot’s orientation on the target position.

## 3. Frontier-Graph Structure

In our previous works, we proposed a method to extract frontier nodes on a local grid map and construct the frontier-tree for local map-based exploration [28,29]. In this paper, we rename the frontier-tree to obtain a frontier-graph that represents the proposed frontier node-edge structure. The frontier nodes and their relative transformations constitute a graph. However, there are several loops, and the nodes have hierarchical relationships: root, parent, and child nodes, unlike in the case of a general graph. We present the tree-like graph structure containing the frontier information.

**Figure 1:**
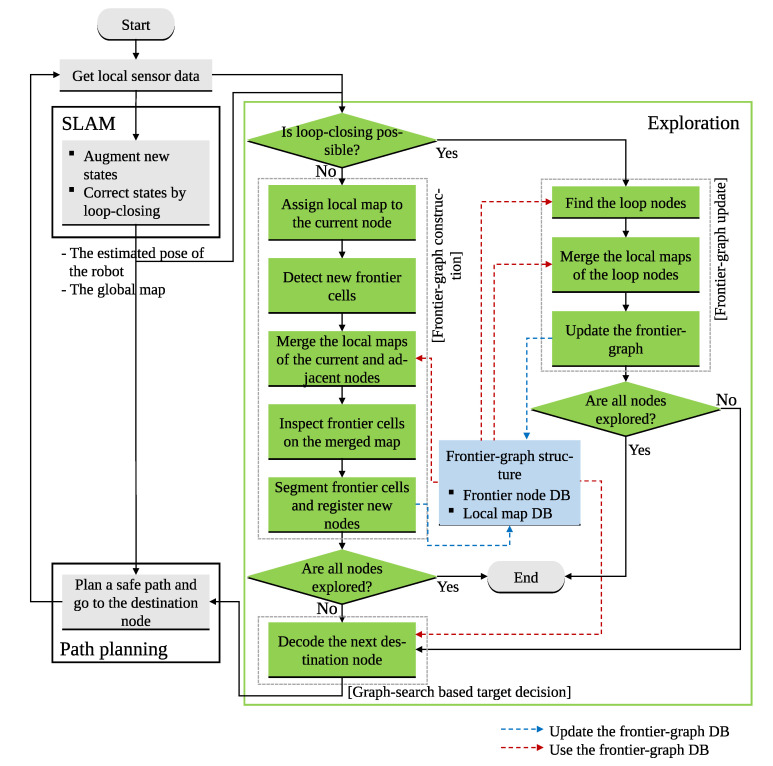
Overview of the proposed integrated exploration algorithm using a frontier-graph structure and graph search-based decision.

In this section, we elaborate on the frontier-graph structure used for local map-based exploration. Figure 1 shows the overall process of the integrated exploration using the frontier-graph structure and the corresponding local maps. The integrated exploration algorithm consists of three sub-processes: SLAM, exploration, and path planning. When local sensor data are obtained, the SLAM algorithm estimates the state vector, which contains the pose of the robot and representation of the environment. Using the estimated pose of the robot and local sensor data, the exploration algorithm detects frontier regions and decides the next destination. Then the robot goes to the next destination along the safe path calculated by the path planning algorithm, until it reaches its destination. In this paper, we assume that the SLAM and path planning algorithm performs adequately to estimate the pose of the robot and steer it to the target position at the desired frequency. We focus on the function of the exploration algorithm that selects the next target destination for expanding the map information or reducing the uncertainty of the robot’s pose by loop-closing.

### 3.1. Constructing Frontier-Graph Structure Using Local Maps

#### 3.1.1. Frontier-Graph Database

From the perspective of the proposed local map-based exploration method for constructing the frontier-graph, exploration is the sequential process of expanding the graph by detecting and adding frontier nodes until new nodes are no longer discovered. Eventually, the entire environment is modeled as distributed nodes and edges between these nodes. Before we present details on the frontier-graph, we define the frontier area and the non-frontier area for the sake of clarity. A frontier area is an edge segment that separates the known (explored) empty regions from unknown (unexplored) regions. It can be an unknown cell next to known empty cells or a known empty cell next to unknown cells. In this paper, the frontier area is defined as a known empty cell next to unknown cells. The position of the frontier area can be the next exploration target, and it needs to be empty so that the robot can arrive there. A non-frontier area is a grid cell that is not a frontier cell. Table 1 shows the components of the frontier node and local grid map database (DB) for the frontier-graph structure. There are two types of DB: frontier node DB (Node) and local map DB (Map). The frontier node DB includes geometric information regarding the frontier segments and exploration state. The local map DB stores local grid map information of each visited frontier node.

Each node, $node_{id}$, has its own identification number (id). If a new representative node of the frontier segment is selected from the current node’s local grid map, the new node is registered to the frontier node DB as the child of the current node. $id_{\mathrm{parent}}$ is the ID of the parent node, and $cells_{id}$ stores the metric position of each frontier cell in $node_{id}$’s segment. Because the newly detected frontier node means that it has not been explored yet, the ID of the local grid map, $id_{\mathrm{map}}$, is initially set to 0. When the robot arrives at the node and obtains the local grid map, the local grid map is added to the local map DB and the corresponding local map ID is assigned to $id_{\mathrm{map}}$ of $node_{id}$. $flag_{\mathrm{known}}$ is the exploration state, which indicates whether the node has been explored or not. It is FALSE when the node is initially registered, and transforms to TRUE after the robot has explored the node or the node has been revealed to be a known area on the merged map. $flag_{\mathrm{loop}-\mathrm{closing}}$ indicates whether a loop has been closed at the node, and is initially set to FALSE. If the robot revisits the node and a loop-closing event occurs, it becomes TRUE. If *n* frontier segments are extracted at the current node, the IDs of the *n* representative cells are stored in $\left\{id_{{\mathrm{child}}_{1}},\cdots ,id_{{\mathrm{child}}_{n}}\right\}$ as child nodes.

The root node is the starting position of the exploration process. If the robot detects *n* frontier segments at the starting position, the information concerning the root node and *n* child nodes is as follows:

(1) $$\begin{array}{c} \mathrm{root}\;\mathrm{node}:\;node_{1}=\left\{1,0,\emptyset ,1,TRUE,FALSE,\left\{2,\cdots ,n+1\right\}\right\} \end{array}$$

(2) $$\begin{array}{ccc} n\;\mathrm{child}\;\mathrm{nodes}:\;node_{2} & = & \left\{2,1,cells_{2},0,FALSE,FALSE,\emptyset \right\},\cdots ,\\ node_{n+1} & = & \left\{n+1,1,cells_{n+1},0,FALSE,FALSE,\emptyset \right\} \end{array}$$

We define the root node’s ID to be 1, and its parent’s ID to be 0. Because the robot essentially starts the exploration process in an empty region, the root node does not appertain to any frontier segments: the third component in Equation (1) is *∅*. $edge_{i,j}$ is the information concerning the relative transformation from $node_{i}$ to $node_{j}$, and consists of the shortest distance, $dist_{i,j}$, between the two nodes and the geometric relative transformation vector, ${\mathrm{tr}}_{i,j}$. A general tree structure only has directed edges between the parent and child nodes. In our frontier-graph structures, all child nodes have one parent node, and there are edges between the parent and child nodes. Moreover, because we can compute the relative transformations between child nodes when new child nodes are detected on the local grid map, there are edges between child nodes, i.e., sibling nodes.

If the robot has visited a frontier node, the local grid map is assigned to the node and this local map, along with its ID, is added to the local map DB. $gridmap_{id}$ of $map_{id}$ contains the grid map data, such as the occupancy probability. $\left\{id_{node_{1}},\cdots ,\;id_{node_{m}}\right\}$ shows the IDs of the nodes that share $map_{id}$, and $\left\{p_{node_{1}},\cdots ,\;p_{node_{m}}\right\}$ are their positions in $map_{id}$. One local map has one node at the time that it is added to the local map DB. If the robot constructs a local map at the current $node_{k}$ and there have already been *q* local maps in the local map DB, the new local map is added as follows:

(3) $$\begin{array}{c} map_{q+1}=\left\{q+1,gridmap_{q+1},\left\{k\right\},\left\{{\left[0,0,0\right]}^{T}\right\}\right\} \end{array}$$

The pose of the node on the local grid map is initially set to ${\left[0,0,0\right]}^{T}$ because the local grid map is constructed at the node. If a loop is closed at the current node, the local maps of nodes that make up the loop will be merged into a larger grid map, and the poses of the loop nodes are corrected on the larger grid map. The map information of the current node, i.e., the loop-closing node, is also updated and contains several nodes and corresponding corrected poses. In other words, the loop nodes with accurate states share the same merged map after loop-closing. An example of the node and map DB before/after loop-closing when the root node has two child nodes is presented in Table 2. The underlined components are the updated components. Notably, the loop-closing state of $node_{1}$, the sixth component, was changed to TRUE.

#### 3.1.2. Frontier Segmentation

If the robot arrives at a new frontier node, the current local grid map is assigned to the current node of the frontier-graph structure. Because the efficiency of the graph-based algorithm depends on the number of nodes, it is crucial to extract real frontier cells and register several representative nodes to the frontier-graph by segmenting real frontier regions. Although conventional frontier-based exploration uses global information from the entire grid map, the proposed local map-based exploration only uses local information to detect frontiers and select the next target. The frontier cells are extracted from the current local grid map. At this time, fake frontier regions, which are frontier regions on the local map that have already been explored so are known regions on the adjacent nodes’ local maps, can be detected. To prevent fake frontier nodes from being registered to the graph and distinguish them from real frontier cells, we merge the local maps of adjacent neighbor nodes. We segment the frontier cells that are frontiers on both the current local map and the merged map.

(4) $$\begin{array}{c} map_{\mathrm{merged}}=map_{\mathrm{current}}\cup map_{\mathrm{current}}^{\mathrm{parent}}\cup map_{\mathrm{current}}^{\mathrm{previous}} \end{array}$$

**Figure 2:**
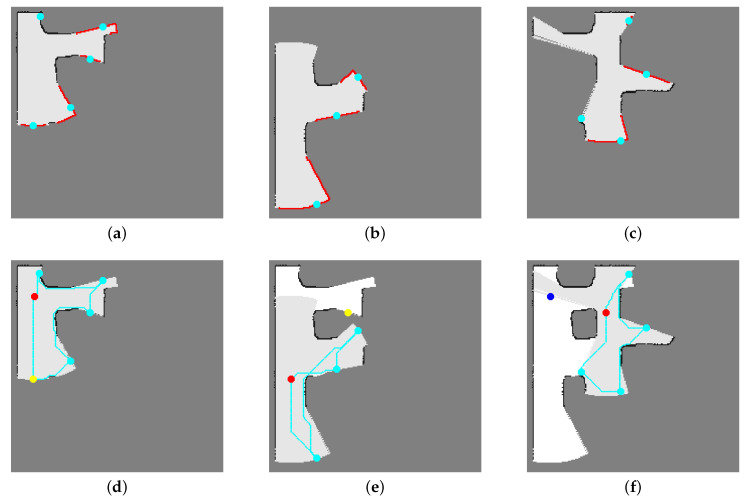
Examples of frontier segmentation. Medium gray indicates unknown cells; black indicates occupied cells; very light gray indicates empty cells; red stars are detected frontier cells; cyan dots are representative nodes; red dots are current nodes; yellow dots are unexplored next targets; the blue dot is the next explored target; cyan lines show the solution to the traveling salesman problem (TSP) for the current node and its child nodes: (**a**) Local map constructed at the root node (**b**) local map constructed at the root node’s first child node (**c**) local map constructed at the root node’s third child node (**d**). A single map is used at the root node to discriminate real frontiers. (**e**) The local maps of the current and parent nodes are merged. The previous node is the parent node. The root/parent node’s second child node is not at the frontier area in the merged map, and is eliminated from the exploration target list to obtain a new observation. (**f**) The local maps of the current, parent, and previous nodes are merged. The previous node is the sibling node.

Three adjacent nodes take part in this map merging operation, as in Equation (4). The local maps of the current, parent, and previous nodes are merged. The previous node is the node that the robot has visited just before arriving at the current node. If the previous node is the parent node, two local maps, the local maps of the current and the parent nodes, are merged. In Equation (4), the reference frame of the merged map is the same as the reference frame of the current local map, and the previous and parent local maps have to be transformed with respect to the pose of the current node. Figure 2 shows how local maps and their merged maps are used to detect real frontier cells. In Figure 2d, the robot is at the root node, and only one grid map is used. Two adjacent local maps, the local maps of the current node and parent node, are merged in Figure 2e. Three adjacent local maps, the local maps of the current node, parent node, and previous node, are merged in Figure 2f.

In our previous works, we constructed a polar histogram of the frontier cells with respect to the current robot pose so that we could segment them. To distinguish the disconnected frontier region, we used a threshold value: the number of empty histogram bins between two frontier cells. If the orientation differences in two adjacent frontier cells are higher than the threshold value, two cells are divided into different segments. However, this previous method does not deal with the case where there is a thin obstacle between frontier regions, and the threshold value depends on the size of a grid cell and the distance to the frontier cell. To overcome these problems, we apply an efficient computer vision approach, the connected components labeling (CCL) algorithm [30]. The CCL algorithm is an image segmentation algorithm that assigns a unique label to all pixels of each connected component in a binary image. We convert the local grid map to a binary image where the frontier cells are 1 and the others are 0. Applying the CCL algorithm with 8-connectivity to the frontier binary image, we can obtain several frontier segments and select the representative frontier cell of each segment.

Algorithm 1 shows the new node registration algorithm by frontier segmentation for when the robot arrives at the newly visited node. We describe the algorithm assuming that the current node is $node_{n}$, the parent node is $node_{n-2}$, and the previous node is $node_{n-1}$. The current node and the previous node are siblings to each other. In other words, they were detected at the parent node, $node_{n-2}$. The map IDs of the parent node and the previous node are M−2 and M−1. At the current node, the current local grid map is constructed and added to the local map DB according to Equation (3), and the local map ID is *M* (line 1). The current local map is merged with the local maps of adjacent nodes in line 2. In line 3, we detect frontier cells in the current grid map, $gridmap_{cur}$. The empty cells adjacent to unknown/unobserved cells are extracted as the frontier cells. The second input argument, window_size, is the range to check the safety against occupied cells by obstacles. The algorithm inspects whether there are any occupied cells within the window centered each frontier candidate position or not. Among the extracted frontier cells of the local grid map, we select the real frontier cells that are also frontiers on the merged map (lines 4–13). The cell position, ${\left[r,c\right]}^{T}$, in the local map is transformed to the position with respect to the merged map frame using the edge DB (line 7), and is inspected by Algorithm 3 (line 8). The real frontier cells are divided into several segments according to their adjacent connectivity using CCL algorithm (line 14). For each frontier segment, we decide the representative frontier node that is the nearest cell to the median of the frontier cell positions in the segment and calculate the edge information about the relative transformation between the current node and the representative cell that becomes a child node (lines 18–22). In addition, we calculate the edge information between the child nodes (line 23), which can be obtained by the composition operator [31], as follows:

(5) $$\begin{array}{c} {\mathrm{tr}}_{child_{1},child_{2}}=⊖{\mathrm{tr}}_{cur,child_{1}}⊕{\mathrm{tr}}_{cur,child_{2}} \end{array}$$

The shortest travel distance, $dist_{i,j}$, among the frontier nodes of Edge, can be determined by the grid-based path-finding algorithm such as A* search algorithm using the local grid map [32].

In our previous works, we added new nodes to the frontier node DB in any order. Here, we propose regisinformation between the child nodestering new nodes according to the solution of the traveling salesman problem (TSP) (line 24). As described in Section 4, we use BFS exploration to select the next target from the frontier nodes, which induces sequential loop-closing from the root node. The robot goes back to the parent node after exploring all child nodes during the “induced” loop-closing, and the nearest child or sibling node was selected as the next target according to the previous method. In this paper, we treat finding an efficient loop-closing path, an efficient exploration order, among the parent and the child nodes as solving a TSP [33,34]. Using the shortest traveling distance, $dist_{i,j}$ in $Edge_{new}$, is the cost matrix of the TSP algorithm, we decide the exploration priority for the current and new nodes. The new nodes are registered to the node DB as the current node’s child nodes in the order of the solution to the TSP (lines 24–26). This means that the higher number of the node’s ID, the higher its priority during the induced loop-closing. Figure 2d–f shows the solutions to the TSP for the current and child nodes (cyan lines).
**Algorithm 1** Registration of new nodes by frontier segmentation**Require:** current local grid map $gridmap_{cur}$, frontier-graph database $Node=\left\{node_{id}|1\leq id\leq N\right\}$, Edge, $Map=\left\{map_{id}|1\leq id\leq M-1\right\}$, size of window for detecting safe frontier cells window_size**Ensure:** updated frontier-graph database, Node,Edge,Map▹ The algorithm assumes that the current node is $node_{n}$, the parent node is $node_{n-2}$, and the previous node is $node_{n-1}$. $node_{n}$ and $node_{n-1}$ are siblings to each other. The map IDs of the parent node and previous node are M−2 and M−1.1: add $map_{M}=\left\{M,gridmap_{M},\left\{M\right\},\left\{{\left[0,0,0\right]}^{T}\right\}\right\}$into Map2: obtain $gridmap_{mg}$ by merging the adjacent local maps, $map_{M-2}$, $map_{M-1}$, $map_{M}$,3: gridmap_frontier←
Detect-Frontier-Cell
$\left(gridmap_{cur},window\_size\right)$   ▹RowSize is the number of rows gridmap_frontier and $gridmap_{cur}$.   ▹ColSize is the number of columns gridmap_frontier and $gridmap_{cur}$.4: **for**
r=1 to RowSize
**do**5:     **for**
c=1 to ColSize
**do**6:         **if**
$gridmap\_frontier\left(r,c\right)=1$
**then**7:            ${\left[r_{mg},c_{mg}\right]}^{T}\leftarrow \mathrm{cell}\_\mathrm{coordinate}\_\mathrm{transform}\left({\left[r,c\right]}^{T},gridmap_{cur},gridmap_{mg},Edge\right)$8:            **if**
Inspect-Safe-Frontier-Cell$\left({\left[r_{mg},c_{mg}\right]}^{T},window\_size,gridmap_{mg}\right)=0$
**then**9:                $gridmap\_frontier\left(r,c\right)\leftarrow 0$10:            **end if**11:         **end if**12:     **end for**13: **end for**
14: $frontier\_segment\leftarrow \mathrm{connected}\_\mathrm{component}\_\mathrm{labeling}\left(grifmap\_frontier\right)$
15: **if**
frontier_segment≠∅
**then**
16:     relative_trans←∅17:     new_Node←∅18:     **for all**
$frontier_{i}\in frontier\_segment$
**do**19:         $\left\{node_{id_{new}},tr_{n,id_{new}}\right\}$←Initialize-New-Node$\left(frontier_{i},n,id_{new}\right)$20:         $new\_Node\leftarrow new\_Node\cup \left\{node_{id_{new}}\right\}$21:         $relative\_trans\leftarrow relative\_trans\cup \left\{tr_{n,id_{new}}\right\}$22:     **end for**23:     $Edge_{new}\leftarrow \mathrm{calculate}\_\mathrm{edge}\left(node_{n},new\_Node,relative\_trans,gridmap_{cur}\right)$24:     $new\_Node\_ordered\leftarrow \mathrm{TSP}\left(node_{n},new\_Node,Edge_{new}\right)$25:     $\mathrm{update}\_\mathrm{node}\_\mathrm{DB}\left(Node,new\_Node\_ordered\right)$26:     $\mathrm{update}\_\mathrm{edge}\_\mathrm{DB}\left(Edge,Edge_{new}\right)$27:
**end if**


Algorithms 2–4, which are used in Algorithm 1, are to detect frontier cells on a grid map, inspect a safe frontier cell without nearby occupied cells, and initialize new node information, respectively. Algorithm 2 first finds empty cells whose occupancy probability is lower than the empty threshold (lines 4–8). By applying the morphological closing operation to the image that presents 1(TRUE) for empty regions and 0(FALSE) for the other regions, we can remove tiny unknown noise cells between empty cells (line 9). We perform morphological erosion with the closed grid map image (line 10). The windows for the morphological operations, wdw_close and wdw_erosion, determine how many noise cells are removed and the width of the internal boundary. We obtain the empty regions’ internal boundary by subtracting the eroded image from the closed image (line 11), and the cells consisting of the internal boundary are frontier candidate cells. The safe frontier cells are obtained by inspecting the nearby occupied cells presented in Algorithm 3 (line 14).
**Algorithm 2** Frontier cell detection**Require:**  
$gridmap=\left\{cell\left(i,j\right)\left|1\leq i\leq RowSize,1\leq j\leq ColSize\right.\right\}$, window_size**Ensure:**  
gridmap_frontier
1: **function**
Detect-Frontier-Cell(gridmap, window_size)2:     set $gridmap\_empty=\left\{empty\left(i,j\right)\left|empty\left(i,j\right)=0,\;1\leq i\leq RowSize,1\leq j\leq ColSize\right.\right\}$3:     set $gridmap\_frontier=\left\{frontier\left(i,j\right)\left|frontier\left(i,j\right)=0,\;1\leq i\leq RowSize,1\leq j\leq ColSize\right.\right\}$4:    **for all**
$cell\left(i,j\right)$
**do**5:        **if**
$cell\left(i,j\right)<th_{\mathrm{empty}}$
**then**6:           $empty\left(i,j\right)\leftarrow 1$7:        **end if**8:    **end for**9:    $gridmap\_empty\_close\leftarrow \mathrm{image}\_\mathrm{morph}\_\mathrm{close}\left(gridmap\_empty,wdw\_close\right)$10:    $gridmap\_empty\_erosion\leftarrow \mathrm{image}\_\mathrm{morph}\_\mathrm{erosion}\left(gridmap\_empty,wdw\_erosion\right)$11:    gridmap_empty_edge←gridmap_empty_close−gridmap_empty_erosion12:    **for all**
$grid\_empty\_edge\left(i,j\right)\in gridmap\_empty\_edge$
**do**13:        **if**
$grid\_empty\_edge\left(i,j\right)=1$
**then**14:           $frontier\left(i,j\right)$←Inspect-Safe-Frontier-Cell$\left({\left[i,j\right]}^{T},window\_size,gridmap\right)$15:        **end if**16:    **end for**17:    **return**
gridmap_frontier18:
**end function**


**Figure 3:**
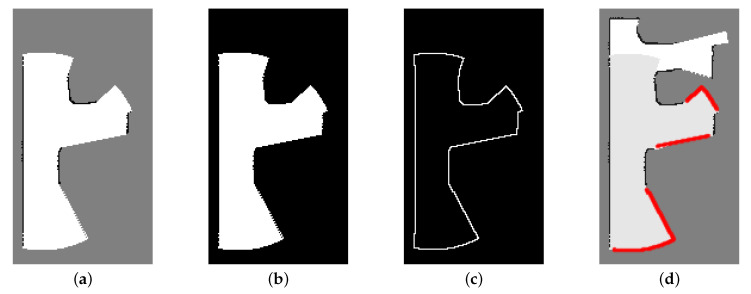
Frontier cell detection: (**a**) Local grid map. (**b**) Empty region of local grid map. (**c**) Internal boundary of empty region extracted by morphological operations. (**d**) Safe frontier cells (red stars) on merged map.

Algorithm 3 counts the occupied and unknown cells around a frontier candidate cell (lines 5–13). If there are no occupied cells and the number of unknown cells is higher than the threshold around one frontier cell, we classify the cell as a safe frontier cell (lines 14–16). Figure 3 shows an example of how to detect safe frontier cells using morphological operations. The safe frontier cells are indicated as red stars in Figure 3d.

Algorithm 4 initializes a new node for each frontier segment. The representative node position that could be the robot’s exploration target position in future steps is determined to be the nearest cell to the median of the frontier segment cells (lines 2–4). The edge information defined by the relative transformation between two nodes is also calculated (line 5). With the metric positions of the frontier segment cells with respect to the current node, the new node is initialized and registered as a child of the current node (lines 6–11).
**Algorithm 3** Safety inspection of a frontier cell**Require:** empty cell position ${\left[r,c\right]}^{T}$, size of window for detecting safe frontier cells window_size, $gridmap=\left\{cell\left(i,j\right)\left|1\leq i\leq RowSize,1\leq j\leq ColSize\right.\right\}$**Ensure:** 
$flag_{\mathrm{frontier}}$
1: **function**
Inspect-Safe-Frontier-Cell(${\left[r,c\right]}^{T}$, window_size, gridmap)2:     $flag_{\mathrm{frontier}}\leftarrow 0$3:     number_of_unknown←04:     number_of_occupied←05:     **for**
$p=r-\mathrm{round}\left(window\_size/2\right)$ to $r+\mathrm{round}\left(window\_size/2\right)$
**do**6:         **for**
$q=c-\mathrm{round}\left(window\_size/2\right)$ to $c+\mathrm{round}\left(window\_size/2\right)$
**do**7:            **if**
$cell\left(p,q\right)>th{}_{\mathrm{occupied}}$
**then**8:                number_of_occupied←number_of_occupied+19:            **else if**
$cell\left(p,q\right)>th{}_{\mathrm{empty}}$
**then**10:                number_of_unknown←number_of_unknown+111:            **end if**12:         **end for**13:     **end for**14:     **if**
number_of_occupied<1**and**$number\_of\_unknown>th_{\mathrm{num}\_\mathrm{unknown}}$
**then**15:         $flag_{\mathrm{frontier}}\leftarrow 1$16:     **end if**17:     **return**
$flag_{\mathrm{frontier}}$18:
**end function**

**Algorithm 4** New node initialization**Require:** 
cell positions of one frontier segment cells_frontier, the current node’s ID $id_{cur}$, a new frontier node’s ID $id_{new}$**Ensure:** 
$node_{id_{new}}$, $tr_{id_{cur},id_{new}}$1: **function**
Initialize-New-Node(cells_frontier, $id_{cur}$, $id_{new}$)2:     ${\left[r_{m},c_{m}\right]}^{T}\leftarrow \mathrm{calculate}\_\mathrm{median}\_\mathrm{position}\left(cells\_frontier\right)$3:     ${\left[r_{rep},c_{rep}\right]}^{T}\leftarrow \underset{{\left[p,q\right]}^{T}\in cells\_frontier}{\arg\min}∥{\left(p-r_{m}\right)}^{2}+{\left(q-c_{m}\right)}^{2}∥$4:     ${\left[x_{rep},y_{rep}\right]}^{T}\leftarrow \mathrm{find}\_\mathrm{metric}\_\mathrm{position}\left({\left[r_{rep},c_{rep}\right]}^{T},gridmap_{cur}\right)$5:     $tr_{id_{cur},id_{new}}\leftarrow {\left[x_{rep},y_{rep},0\right]}^{T}$6:     $cells_{id_{new}}\leftarrow \emptyset$7:     **for all**
${\left[p,q\right]}^{T}\in cells\_frontier$
**do**8:         ${\left[x,y\right]}^{T}\leftarrow \mathrm{find}\_\mathrm{metric}\_\mathrm{position}\left({\left[p,q\right]}^{T},gridmap_{cur}\right)$9:         $cells_{id_{new}}\leftarrow cells_{id_{new}}\cup \left\{{\left[x,y\right]}^{T}\right\}$10:     **end for**11:     $node_{id_{new}}\leftarrow \left\{id_{new},id_{cur},cells_{id_{new}},0,FALSE,FALSE,\emptyset \right\}$12:     **return**
$\left\{node_{id_{new}},\;tr_{id_{cur},id_{new}}\right\}$13:
**end function**


### 3.2. Updating Frontier-Graph Structure by Loop-Closing

We register new frontier nodes as child nodes to the frontier-graph structure when the robot arrives at the previously unexplored node and detects safe frontier cells. We update the node and local map DB if the SLAM algorithm corrects the edge information by loop-closing. The SLAM algorithm deals with detecting the loop-closing event and the consequent state update, and is beyond the scope of this paper. We focus on how to update the proposed frontier-graph structure after loop-closing.

There are two types of loop-closing events: induced loop-closing and accidental loop-closing. Induced loop-closing is intentionally performed using the exploration algorithm to select the next target. Accidental loop-closing is caused by the property of the local map-based approach, namely that the robot only depends on adjacent local information to inspect the exploration state of the nodes, whether explored or not. This is the case when the robot revisits the same node by chance. The robot does not realize that the target node has already been explored or is very close to a previously visited node.

In induced loop-closing from a child node to its parent node, the loop sequence is distinct. We can obtain the node hierarchy from the frontier-graph, and the sequence is composed of the parent node and explored child nodes, as follows:

(6) $$\begin{array}{c} node_{\mathrm{parent}}\to node_{{\mathrm{child}}_{1}}\to node_{{\mathrm{child}}_{2}}\to \cdots \to node_{{\mathrm{child}}_{n}}\to node_{\mathrm{parent}} \end{array}$$

We can obtain the loop constraint from the edge information of the node DB, and the SLAM algorithm optimizes and corrects the corresponding states according to the constraint. Based on the corrected edge information, the local maps are merged into a larger map, and the loop nodes are computed with respect to the parent node.

In accidental loop-closing, we first must find a sequence that is not composed of just a parent node and its child nodes. To do this, we find a path from the revisited node (the start node) to the previous node (the target node) using the edge information of the node DB and a shortest path algorithm for graphs, such as Dijkstra’s algorithm. This path, together with the current node, makes the following loop sequence:

(7) $$\begin{array}{c} node_{\mathrm{revisited}}\to node_{s_{1}}\to \cdots \to node_{s_{n}}\to node_{\mathrm{previous}}\to node_{\mathrm{current}}\to node_{\mathrm{revisited}} \end{array}$$

The edge between the current node and the revisited node is acquired from SLAM’s loop-closure detection. We update the edge and local maps of the loop nodes in the same manner as induced loop-closing. We also register the edges between the current and revisited nodes’ neighbor nodes.

**Figure 4:**
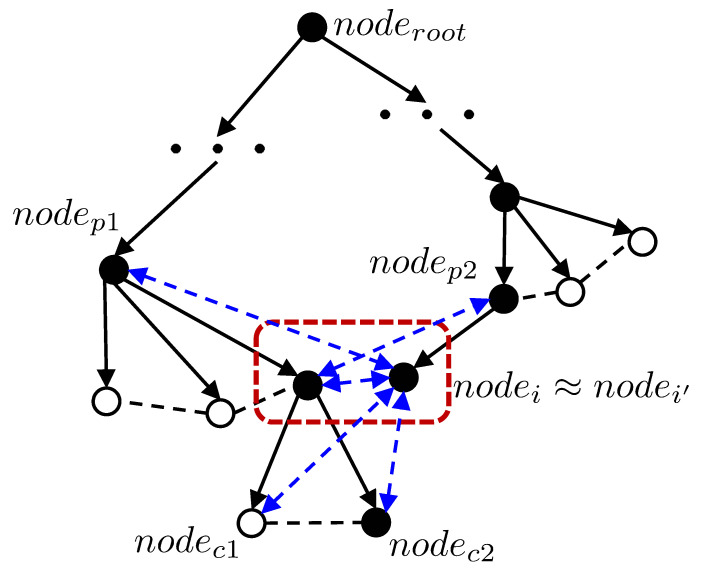
Registering the edges between the current and revisited nodes’ neighbors in the frontier node DB after accidental loop-closing.

Figure 4 shows the updated edges after accidental loop-closing. Considering the fact that the current and revisited nodes are close to each other, it is reasonable to think that the current node and the revisited node share the same parent and child nodes. In Figure 4, we generate the edge that links the parent node ($node_{p2}$) of the current node ($node_{i^{\prime }}$) and the revisited node ($node_{i}$). The parent node ($node_{p1}$) of the revisited node and the current node are connected. Edges between the current node and the revisited node’s children are created. Using this additional edge registration, two parts that are distant from each other in the graph structure, but close together in the geometric structure, will be connected.

## 4. Graph-Search-Based Exploration for Frontier-Graph Structure

One of the essential aspects of exploration is to efficiently select the next target according to priority. We can recognize exploration priority by inspecting the exploration state and adjacency of the frontier-graph structure’s nodes. We can induce a loop-closing event by going back to a previously explored adjacent node or making the robot go to the nearest unexplored node by traversing the edges of the graph.

In the BFS exploration, unexplored sibling nodes have a higher priority than child nodes [29]. If all sibling nodes have been explored and have local maps, the robot goes back to the parent node for loop-closing. Until then, BFS exploration prefers to improve the accuracy and consistency between the registered nodes rather than add new nodes to expand the mapped area. However, the DFS selects the nearest unexplored child node as the next target until the robot reaches a leaf node that has no child nodes [28]. When new frontier nodes are not detected at the current node, the robot returns to the parent node. This means that the number of nodes increases until we arrive at the leaf node. In simple corridor environments with closed walls at both ends, the DFS performs efficiently because the walls become leaf nodes. However, in complex or large environments, the efficiency of DFS-based exploration depends on the loop-detection performance of SLAM. If no opportunity for accidental loop-closing is detected, updating the node DB and merging the local maps are delayed, and there is a higher probability of adding fake frontier nodes. This issue becomes severe where there is a structural loop in the environment. The robot continuously adds new nodes while traveling multiple times around the loop, until loop-closing occurs by chance. To efficiently manage the number of frontier nodes according to the consecutive map merging from the beginning of the exploration process, we improve BFS exploration in this paper.

The previous BFS exploration algorithm selects the nearest unexplored child node after exploring the parent node, or selects the nearest unexplored sibling node before induced loop-closing to the parent node, i.e., the shortest traveling distance plays a critical role in deciding the next target node. In this paper, we applied the TSP solution to register new child nodes in Section 3.1.2. This is used to find an efficient loop-closing sequence, and we improve BFS-based exploration using the loop-closing sequence obtained from the solution to the TSP.

Algorithm 5 shows the improved BFS exploration using the solution to the TSP. First, the robot inspects whether there are unexplored siblings or not (line 1) using the component $flag_{\mathrm{known}}$ in the node DB. If unexplored sibling nodes are recognized, the robot selects the next node from these. At this time, among several unexplored siblings, the node next to the current node along the node sequence obtained from the solution to the TSP is selected (line 3). If all siblings have been explored, we consider the loop-closing state $flag_{\mathrm{loop}-\mathrm{closing}}$ of the parent node. If loop-closing has not been completed at the parent node, the robot goes to the parent node to close the loop composed of the parent, current, and sibling nodes (lines 4 and 5). The fact that loop-closing has not been performed at the current node means that there have not been sufficient measurements to make loop-constraints between the current node and the child nodes. Therefore, the robot goes to one of the unexplored child nodes to obtain measurements and local maps (line 7). Next, we check the loop-closing state of the siblings and child nodes. The corresponding un-loop-closed node is selected as the next target according to the solution of the TSP (lines 10–16). If loop-closing has been performed at all adjacent nodes and all adjacent nodes’ local maps are merged, the robot goes to the nearest unexplored node (line 20). When there is no unexplored node in the frontier-graph, the exploration process is considered complete.

Algorithm 6 describes a method for finding the next target node of the reference/current node from the TSP solution among adjacent nodes. Because we added new child nodes to the frontier-graph DB in the order of the TSP solution, which makes a loop consisting of adjacent nodes, we can determine the traveling priority between the adjacent nodes through each node’s ID. We arrange the adjacent nodes, NodeTSP, in the ascending order of each node’s ID (line 2). Next, we find the position of the reference node in the sorted array (line 3). The next target node of the TSP node sequence is at the position next to the reference node. If the reference node’s position is the end of the array, the next node is the first node of the array, because these compose the loop (lines 4–8).
**Algorithm 5** Breadth-first search (BFS) exploration to decide the next target node**Require:** node DB Node, the current node’ ID $id_{cur}$, local map DB Map**Ensure:** 
the next target node $node_{next}$1:
$Node_{unexp\_sibling}\leftarrow \mathrm{find}\_\mathrm{unexplored}\_\mathrm{siblings}\;\left(Node,id_{cur}\right)$
2: **if**
$Node_{unexp\_sibling}\neq \emptyset$
**then**
3:     $node_{next}\leftarrow$Find-Next-Node-In-TSP-Solution$\left(Node_{unexp\_sibling},id_{cur}\right)$4:
**else if**
$\left(flag_{\mathrm{loop}\_\mathrm{closing}}\in node_{parent}\right)=FALSE$
**then**
5:     $node_{next}\leftarrow node_{parent}$6: **else if**
$\left(flag_{\mathrm{loop}\_\mathrm{closing}}\in node_{cur}\right)=FALSE$
**then**
7:     $Node_{unexp\_child}\leftarrow \mathrm{find}\_\mathrm{unexplored}\_\mathrm{child}\left(Node,id_{cur}\right)$8:    $node_{next}\leftarrow$Find-Next-Node-In-TSP-Solution$\left(Node_{unexp\_child},id_{cur}\right)$9:
**else**
10:     $Node_{unLC\_sibling}\leftarrow \mathrm{find}\_\mathrm{unLoopClosed}\_\mathrm{siblings}\left(Node,id_{cur}\right)$11:     **if**
$Node_{unLC\_sibling}\neq \emptyset$
**then**12:         $node_{next}\leftarrow$Find-Next-Node-In-TSP-Solution$\left(Node_{unLC\_sibling},id_{cur}\right)$13:     **else**14:         $Node_{unLC\_child}\leftarrow \mathrm{find}\_\mathrm{unLoopClosed}\_\mathrm{child}\left(Node,id_{cur}\right)$15:         **if**
$Node_{unLC\_child}\neq \emptyset$
**then**16:            $node_{next}\leftarrow$Find-Next-Node-In-TSP-Solution$\left(Node_{unLC\_child},id_{cur}\right)$17:         **else**18:            $Node_{unexp}\leftarrow \mathrm{find}\_\mathrm{unexplored}\left(Node\right)$19:            **if**
$Node_{unexp}\neq \emptyset$
**then**20:                $node_{next}\leftarrow \mathrm{find}\_\mathrm{nearest}\left(Node_{unexp},id_{cur}\right)$21:            **else**
22:                $node_{next}\leftarrow 0$▹ The exploration has been completed.23:            **end if**24:         **end if**25:     **end if**26:
**end if**

**Algorithm 6** Next node selection in the solution to the traveling salesman problem (TSP)**Require:** $Node_{TSP}$, the reference node’s ID $id_{Ref}$**Ensure:** $node_{next}$1: **function**
Find-Next-Node-In-TSP-Solution($Node_{TSP}\cup node_{Ref}$)2:     $Node_{ordered}\leftarrow \mathrm{sort}\_\mathrm{node}\_\mathrm{ascend}\left(Node_{TSP}\right)$3:     $pos_{Ref}\leftarrow \mathrm{find}\_\mathrm{node}\_\mathrm{position}\_\mathrm{in}\_\mathrm{node}\_\mathrm{array}\left(Node_{ordered},id_{Ref}\right)$4:     **if**
$pos_{Ref}<N$
**then**▹*N*: the number of components in $Node_{ordered}$5:         $node_{next}\leftarrow {\left[pos_{Ref}+1\right]}^{\mathrm{th}}$ component of $Node_{ordered}$6:     **else**7:         $node_{next}\leftarrow$ the first component of $Node_{ordered}$8:     **end if**9:     **return**
$node_{next}$10: **end function**


## 5. Simulation Results

We simulated our BFS-based exploration algorithm to verify that it efficiently manages frontier information and merges the local maps assigned to the frontier position from the root node, until the robot has explored the whole environment. We compare BFS- and DFS-based exploration in terms of the nodes’ state in the frontier-graph structure.

**Figure 5:**
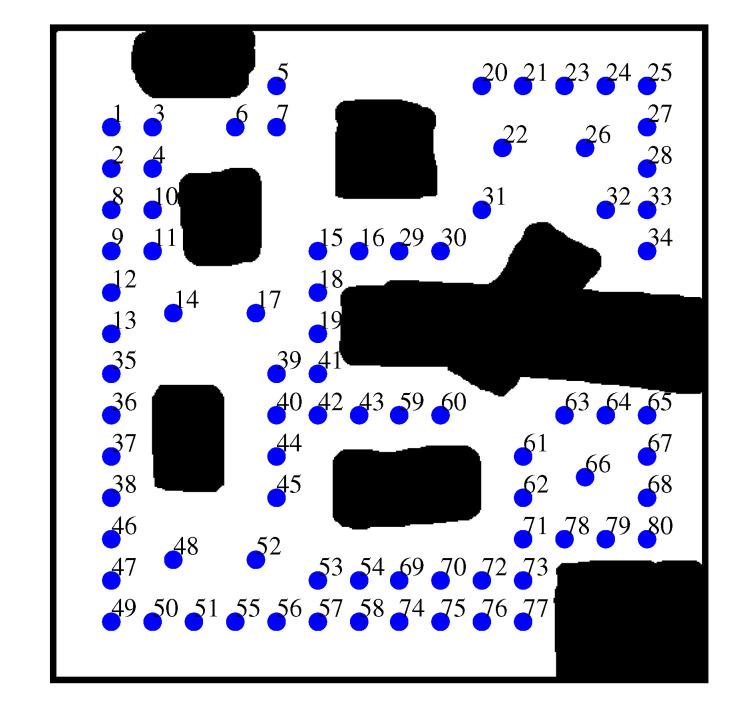
The environment and 80 starting positions.

We used a publicly available cave-like environment [35] for this comparison. All the simulations were performed in Matlab. We assumed that the robot has a laser range finder (LRF) that scans in all directions (360${}^{\circ }$) around the robot. We assume that the SLAM algorithm gives the robot’s state in the global reference frame at every decision step in all simulations, because we focus on the exploration method itself in this paper. However, we only use the SLAM global state to calculate the relative transformation between the frontier nodes. Our local map-based exploration does not depend on SLAM’s global information, and is performed using local information about the frontier nodes, which will be merged incrementally as the exploration proceeds. To merge the local grid maps, the overlapped regions are updated using Bayes’ rule [36]. Figure 5 shows the environment used in the simulations. The robot started the exploration process at 80 different positions (blue dots) for each exploration method. The starting positions were extracted by quadtree cell decomposition [37]. The size of the environment was 20 × 20 m, and it was simulated as a 500 × 500-pixel array. Each local map was constructed using grid cells of 0.1 × 0.1 m size.

**Figure 6:**
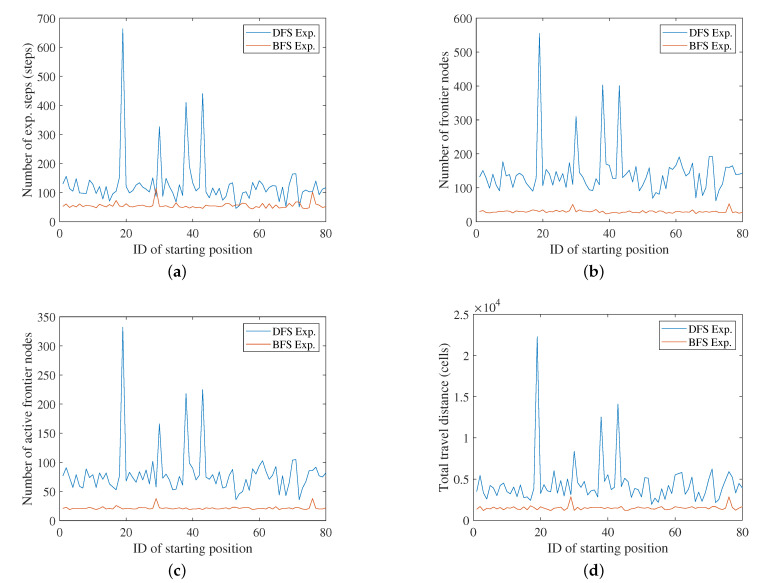
Simulation results. Maximum range of the LRF is 4 m: (**a**) Number of exploration steps until the exploration is completed. (**b**) Total number of registered frontier nodes in the frontier-graph. (**c**) Number of active nodes. (**d**) Total traveling distance.

**Figure 7:**
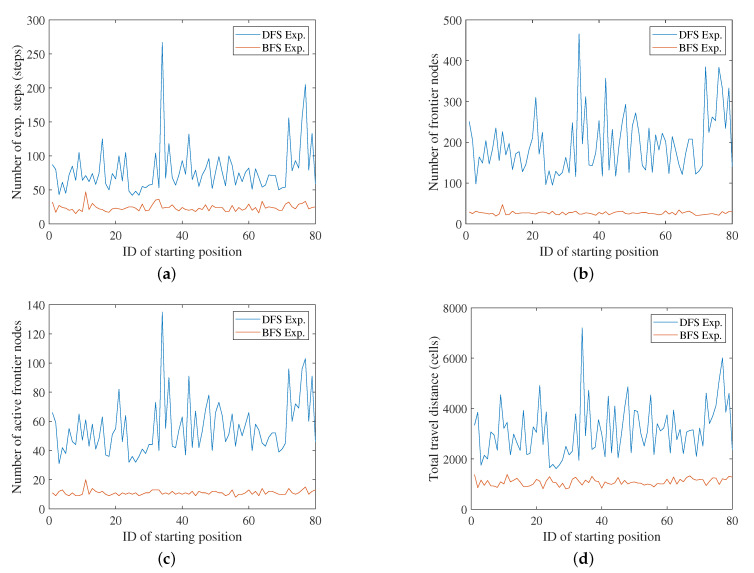
Simulation results. Maximum range of the LRF is 8 m: (**a**) Number of exploration steps until completion of exploration. (**b**) Total number of registered frontier nodes in the frontier-graph. (**c**) Number of active nodes. (**d**) Total traveling distance.

Figure 6 and Figure 7 compare the results of the DFS- and BFS-based exploration. Table 3 and Table 4 are the corresponding statistical results. We compared both methods in terms of four criteria. The first is the number of exploration (exp.) steps, which indicates how many steps/decisions were performed until the entire environment had been mapped. The fewer exploration steps needed, the more efficient the exploration method. Next, we compared the number of registered nodes in the frontier-graph structure. Because the local map-based exploration continues its mapping process until there are no unexplored frontier nodes in the graph, it is crucial to detect and register well-distributed frontier nodes to cover the entire environment. If too many nodes have been registered to the graph, then there are repetitive nodes at similar positions, which makes the exploration process inefficient. Active nodes are the frontier nodes that have been actually explored/visited by the robot among the registered frontier nodes of the frontier-graph structure. The number of active nodes is the same as the number of local maps or measurements used for the entire mapping. The total travel distance is the sum of the distance that the robot went along while traversing the shortest path between the current node and the next node.

As shown in Figure 6 and Figure 7, almost every value for BFS exploration is lower than the corresponding value for DFS exploration, except in very few simulations. The difference between the BFS’s results and the DFS’s results was larger in the case of 8 m LRF than 4 m LRF. Moreover, BFS is more stable than DFS for different starting positions. This means that the efficiency of DFS exploration is critically affected by where the robot starts its exploration process. This is because the local map merging by loop-closing is delayed until the leaf node and multiple frontier nodes can be detected at a similar position in the DFS exploration. The environment is not a simple corridor, which made the exploration inefficient before the accidental loop-closing event.

Table 3 and Table 4 explicitly show the effects of varying the starting position for each method. The ratio of the maximum value to the minimum value ranges from 9.0 to 14.4 in the case of DFS-based exploration with 4 m LRF. The DFS exploration’s maximum travel distance is extremely high, 22,278 cells. However, the ratio of the maximum value to the minimum value ranges from 2.0 to 2.6 in the case of BFS. this is relatively low compared to that of DFS-based exploration. In the case of the results from 8 m LRF, the maximum-minimum ratio is in the range 4.4–6.4 for DFS, while it is in the range 1.7–3.1 for BFS.

The robustness of the BFS exploration is validated based on the standard deviation (SD). The SD of the DFS exploration is much higher than that of the BFS exploration. Because the DFS results are very different for each simulation, we need to select the starting position very carefully to use DFS for exploration. However, this does not coincide with the general mapping assumption or situation, in which the process starts in an unknown environment. The SD of the BFS exploration is low, which implies that we can efficiently explore the environment from any starting position using BFS-based exploration. The local maps of the frontier nodes are systematically merged from the root node, and the merged map is critical for deciding whether each locally detected frontier cell is valuable or not. This prevents a fake frontier region in the local map being registered to the frontier-graph as an exploration target. Therefore, BFS-based exploration proceeds under a stable graph structure, in terms of the number of frontier nodes.

**Figure 8:**
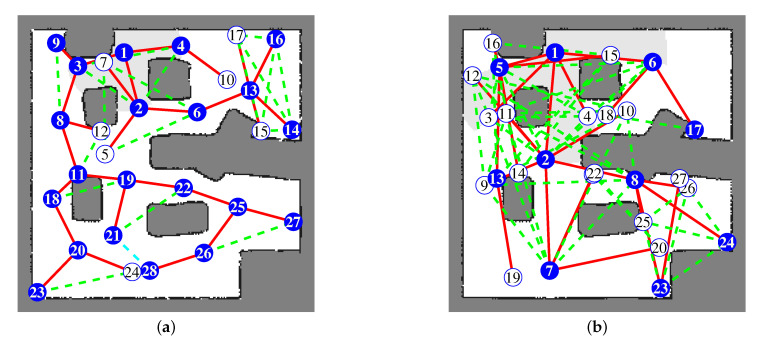
Resulting frontier-graph structure on the final grid map by the BFS exploration. The number is the ID of each frontier node. Red lines are edges between the parent and child nodes. Dotted green lines are edges between sibling nodes. Blue circles are active nodes, and white circles are inactive nodes: (**a**) Frontier-graph from 4 m LRF. (**b**) Frontier-graph from 8 m LRF.

**Figure 9:**
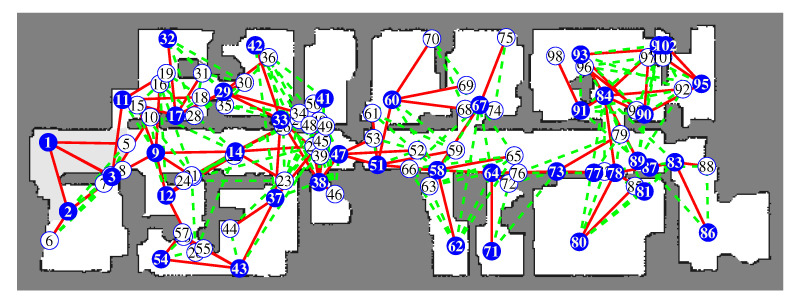
Frontier-graph structure on the final grid map obtained from BFS exploration. The numbers indicate the ID of each frontier node. Red lines are edges between the parent and child nodes. Dotted green lines are edges between sibling nodes. Blue circles are active nodes, and white circles are inactive nodes.

Figure 8 shows the resulting frontier-graph structure. The numbers indicate the ID of each frontier node, and ID-1 is the root node and starting position. The starting positions are the same in both simulations. The red lines show the edges between the parent and child nodes. The dotted green lines indicate the edges between sibling nodes. The blue circles are the active frontier nodes, at which the robot arrived and obtained local maps. The white circles are the nodes that were registered to the frontier-graph, but the robot did not visit. In both results, the entire environment is represented by well-distributed frontier nodes and edges between them. We also carried out simulations using another benchmark environment, Freiburg 079 building [35]. This environment is composed of a corridor and several rooms, and its size is 40 × 13.8 m. The 4 m LRF was used, and the grid map was constructed from 0.07 × 0.07 m grid cells. We can see that the frontier nodes are distributed throughout the environment in Figure 9.

## 6. Conclusions

This paper describes a graph search-based exploration method using frontier detection and segmentation. The proposed method can overcome the difficulty of updating a single global grid map that depends on the exploration state or SLAM in conventional exploration methods. The detected frontier segments on each local grid map and their relative transformations constitute a frontier-graph structure. Because the frontier-graph structure systematically contains the frontier information for the growing map, the robot can gradually construct a map of its environment by traveling along the edges between the frontier nodes.

To select the next exploration target from adjacent frontier nodes, we applied BFS-based exploration that induces incremental map merging by loop-closing from the root node, which is the starting position. Because the proposed method uses only adjacent local information to detect frontiers, BFS is more efficient for exploration than DFS. We verified that BFS-based exploration could achieve more stable results, regardless of the starting position, than DFS-based exploration by carrying out simulations using benchmark environments. Therefore, we can efficiently map unknown environments using the proposed BFS-based exploration method with a frontier-graph structure.

In this paper, we assumed the robot could obtain range measurement in all 360${}^{\circ }$ directions. The field of view of sensors definitely affects exploration efficiency. The reduced field of view sensors can be used for the proposed approach, but the travel distance and the number of active nodes may increase. Taking into account the sensing coverage according to the robot’s orientation will be a valuable future work for a wide range of practical applications.

## Figures and Tables

**Table 1:** Frontier node database and local map database of the frontier-graph structure.

Frontier node DB:
$Node=\left\{node_{id}|1\leq id\leq N\right\}$
$node_{id}=\left\{id,\;id_{\mathrm{parent}},\;cells_{id},id_{\mathrm{map}},\;flag_{\mathrm{known}},\;flag_{\mathrm{loop}-\mathrm{closing}},\;\left\{id_{{\mathrm{child}}_{1}},\cdots ,id_{{\mathrm{child}}_{n}}\right\}\right\}$
$cells_{id}=\left\{{\left[x_{1},y_{1}\right]}^{T},\cdots ,{\left[x_{f},y_{f}\right]}^{T}\left|\begin{array}{c} \mathrm{the}\;\mathrm{metric}\;\mathrm{position}\;\mathrm{of}\;\mathrm{each}\;\mathrm{frontier}\;\mathrm{cell}\;\mathrm{in}\;node_{id}{}^{\prime }s\\ \mathrm{frontier}\;\mathrm{segment}\;w.r.t.\;node_{id} \end{array}\right\}\right.$
$Edge=\left\{edge_{i,j}|\;\mathrm{the}\;\mathrm{relative}\;\mathrm{transformation}\;\mathrm{from}\;node_{i}\;\mathrm{to}\;node_{j}\right\}$
$edge_{i,j}=\left\{{dist}_{i,j},\;{\mathrm{tr}}_{i,j}\right\}$
Local map DB:
$Map=\left\{map_{id}|1\leq id\leq M\right\}$
$map_{id}=\left\{id,\;gridmap_{id},\;\left\{id_{node_{1}},\cdots ,\;id_{node_{m}}\right\},\;\left\{p_{node_{1}},\cdots ,\;p_{node_{m}}\right\}\right\}$
*N*: the number of frontier nodes
*n*: the number of child nodes
*f*: the number of frontier cells in $node_{id}$’s frontier segment
*M*: the number of local maps
*m*: the number of frontier nodes that share $map_{id}$
$p_{node_{m}}$: the pose of $node_{m}$ in the local map $map_{id}$
${\mathrm{tr}}_{i,j}$: ${\left[x_{i,j},\;y_{i,j},\;\theta_{i,j}\right]}^{T}$
$dist_{i,j}$: the shortest distance between $node_{i}$ and $node_{j}$

**Table 2:** An example of a frontier node and local map DB before/after loop-closing.

Before loop-closing:
$node_{1}=\left\{1,0,\emptyset ,1,TRUE,FALSE,\left\{2,3\right\}\right\},\;map_{1}=\left\{1,gridmap_{1},\left\{1\right\},\left\{{\left[0,0,0\right]}^{T}\right\}\right\}$
$node_{2}=\left\{2,1,cells_{2},2,TRUE,FALSE,\left\{id_{\mathrm{chil}d_{2\_1}},\cdots ,id_{\mathrm{chil}d_{2\_n}}\right\}\right\},$
$map_{2}=\left\{2,gridmap_{2},\left\{2\right\},\left\{{\left[0,0,0\right]}^{T}\right\}\right\}$
$node_{3}=\left\{3,1,cells_{3},3,TRUE,FALSE,\left\{id_{\mathrm{chil}d_{3\_1}},\cdots ,id_{\mathrm{chil}d_{3\_n}}\right\}\right\},$
$map_{3}=\left\{3,gridmap_{3},\left\{3\right\},\left\{{\left[0,0,0\right]}^{T}\right\}\right\}$
After loop-closing:
$node_{1}=\left\{1,0,\emptyset ,1,TRUE,\underline{TRUE},\left\{2,3\right\}\right\},\;map_{1}=\left\{1,gridmap_{1},\underline{\left\{1,2,3\right\}},\underline{\left\{{\left[0,0,0\right]}^{T},p_{2},p_{3}\right\}}\right\}$
$node_{2}=\left\{2,1,cells_{2},\underline{1},TRUE,FALSE,\left\{id_{\mathrm{chil}d_{2\_1}},\cdots ,id_{\mathrm{chil}d_{2\_n}}\right\}\right\},\;map_{2}=\underline{\emptyset }$
$node_{3}=\left\{3,1,cells_{3},\underline{1},TRUE,FALSE,\left\{id_{\mathrm{chil}d_{3\_1}},\cdots ,id_{\mathrm{chil}d_{3\_n}}\right\}\right\},\;map_{3}=\underline{\emptyset }$

**Table 3:** Simulation results for 4 m LRF in Figure 6.

	DFS Exp.			BFS Exp.		
	Min.	Avg. (Std.)	Max.	Min.	Avg. (Std.)	Max.
# of exp. steps	46	128.4 (85.9)	663	44	55.6 (10.4)	115
# of frontier nodes	62	143.9 (72.5)	555	23	29.9 (4.5)	53
# of active nodes	36	80.9 (41.1)	332	19	21.5 (3.0)	38
Travel distance (cells)	1929	4401.8 (2754.8)	22278	1173	1500.3 (258.4)	2852

**Table 4:** Simulation results for 8 m LRF in Figure 7.

	DFS Exp.			BFS Exp.		
	Min.	Avg. (Std.)	Max.	Min.	Avg. (Std.)	Max.
# of exp. steps	42	78.3 (35.3)	267	15	23.7 (5.2)	47
# of frontier nodes	95	194.9 (74.2)	466	19	26.3 (3.9)	47
# of active nodes	31	55.6 (18.6)	135	8	11.0 (1.7)	20
Travel distance (cells)	1617	3142.4 (1054.8)	7203	809	1082.8 (139.2)	1383
